# The association between periodontal conditions, inflammation, nutritional status and calcium-phosphate metabolism disorders in hemodialysis patients

**DOI:** 10.1590/1678-7757-2017-0495

**Published:** 2018-07-19

**Authors:** Marta Cholewa, Katarzyna Madziarska, Malgorzata Radwan-Oczko

**Affiliations:** 1 Wroclaw Medical University, Faculty of Medicine and Dentistry, Department and Division of Oral Pathology, Wroclaw, Poland.; 2 Wroclaw Medical University, Faculty of Postgraduate Medical Training, Department and Clinic of Nephrology and Transplantation Medicine, Wroclaw, Poland

**Keywords:** Periodontium, Hemodialysis, Inflammation, Malnutrition

## Abstract

**Objectives:**

To analyze the association between periodontal conditions and inflammation, nutritional status and calcium-phosphate metabolism disorders in hemodialysis (HD) patients.

**Material and Methods:**

We analyzed 128 HD patients divided into two groups: dentate (n = 103) and edentulous (n=25). The following items were assessed: baseline characteristics, age at the start and duration of HD, biochemical data: C-reactive protein (CRP), serum albumin, calcium, phosphorus, alkaline phosphatase, parathormone. A single dentist performed a complete dental/periodontal examination, including parameters of oral hygiene and gingival bleeding.

**Results:**

One person had healthy periodontium, 62.14% of the patients had gingivitis, and 36.9% had moderate or severe periodontitis. The age at HD onset had a positive impact on periodontal status and negatively correlated with the number of teeth. A positive correlation between age and CRP level and negative correlations between age and serum albumin and phosphorus were found. Pocket depth (PD) was negatively correlated with serum albumin. The number of teeth was negatively correlated with serum CRP.

**Conclusions:**

High prevalence and severity of periodontal disease are observed in hemodialysis patients. There is a high probability that periodontal disease may be present at the early stages of chronic kidney disease (CKD) before the hemodialysis onset.

## Introduction

The prevalence of chronic kidney disease (CKD) is an important public health problem worldwide. The loss of renal function is the most serious clinical outcome of CKD^1^. Treatment options include dialysis (hemodialysis or peritoneal dialysis) and/or kidney transplant^2^.

The consequences of undergoing hemodialysis can be the development of several fluid and electrolyte balance disorders (such as volume overload, hyperkalemia, metabolic acidosis, hyperphosphatemia), as well as abnormalities related to hormonal or systemic dysfunctions (such as anorexia, nausea, vomiting, fatigue, hypertension, anemia, bleeding disorders, infection malnutrition, dyslipidemia, bone disease, cardiovascular complications, disturbed thyroid physiology, sexual dysfunction). Patients who are on hemodialysis have higher all-cause and cardiovascular morbidity and mortality associated with traditional (diabetes, hypertension, dyslipidemia) and non-traditional risk factors of atherosclerosis (inflammation and malnutrition)^3^.

The causes of inflammation in hemodialyzed patients are multifactorial. They include poor oral hygiene and worse dental and periodontal state^4^ ^,^ ^8^.

Oral manifestations include halitosis, decrease in salivary secretion – so thirst, xerostomia, increase in salivary pH and salivary urea concentration, uremic stomatitis, calcification of the pulp chamber^5^.

Xerostomia, the subjective feeling of a dry mouth, is relatively common in hemodialyzed patients and is associated with difficulties in chewing, swallowing, tasting and an increased risk of oral disease (infections, dental caries, periodontal disease). This condition can be caused by the use of certain medications, restriction of fluid intake and old age^6^.

There are inconclusive outcomes regarding the prevalence of caries in hemodialysis patients.

In contrast, most studies describe a high incidence of periodontal disease in these patients, where poor oral hygiene with increased deposits of plaque and calculus, identifying the presence of gingival inflammation, deep periodontal pockets, clinical attachment level loss and bone loss^5^ ^,^ ^7^. Furthermore, the dental health status got worse with the duration of hemodialysis treatment^8^.

Borawski, et al.^9^, (2007) found a more severe level of periodontitis in hemodialysis patients than in predialysis chronic kidney disease and peritoneal dialysis patients and finally, more advanced periodontitis in these investigated groups when compared to the control group.

Chronic periodontitis is an inflammatory, multifactorial disease of the periodontal tissues, in which the presence of periopathogens in microbial biofilm is the main initial factor. Moreover, both the quantity and virulence of the periopathogens and host-related general and local risk factors are determinants of the start and progression of periodontal inflammation and loss of periodontal tissue. The increased level of Gram-negative microorganisms responsible for the development and progression of periodontal inflammation increases the immunological response to bacterial antigens^7^ ^,^ ^10^. This may be the link between periodontal disease and CKD due to a concomitant infection and inflammation. A study found that the periodontitis in CKD patients was severer, with increased frequency in the presence of periopathogens and showed a strong association with kidney disease^11^. Periodontal disease is prevalent in kidney failure patients and it can also be, though not always, observed in this group because of the lack of interdisciplinary care for these patients^12^.

The objective of this study was to analyze the association between periodontal conditions and inflammation, nutritional status and calciumphosphate metabolism disorders in hemodialysis patients.

## Material and methods

The patients were recruited in two dialysis centers in Lower Silesia (Poland) – in the Department of Nephrology and Transplantation Medicine of the Wroclaw Medical University and in the Specialist Hospital in Walbrzych.

The study group consisted of 128 patients undergoing hemodialysis (HD). Following dental examination, two groups of patients were formed, dentate patients (n=103) and edentulous patients (n = 25), to find a possible association between periodontal status and the investigated parameters.

The patients included in the study had no history of malignancy, active infections, or diseases requiring immunosuppressive therapy.

All HD patients were dialyzed using a native arteriovenous fistula with adequacy of dialysis — Kt/V≥1.3.

Demographic and clinical data were retrieved from the patient's clinical documentation.

The impact of the following factors on periodontal status were assessed: baseline characteristics (age, sex, race – all Caucasian), age at the start and duration of hemodialysis. We also analyzed biochemical markers of inflammation [C-reactive protein (CRP)], nutritional status (serum albumin) and calcium-phosphate metabolism [serum calcium levels (sCa), phosphorus (sP), alkaline phosphatase (sAP), parathormone (PTH)].

A single dentist performed a complete dental/ periodontal examination, including the parameters of oral hygiene and gingival bleeding.

Dental assessment was performed before HD to exclude the effect of heparin, routinely used during the HD procedure.

Before starting the examination, the dentist was calibrated by a specialist to perform the periodontal assessment. The clinical evaluation comprised the total number of teeth and oral hygiene, according to the Lange^13^ (1986) approximal plaque index (API%) and gingival inflammation according to the Ainamo and Bay^14^ (1975) bleeding on probing index (BoP%). Pocket depth (PD; mm) was determined at four sites of each tooth. Finally, the periodontal status was assessed according to the classification of Offenbacher, Barros and Beck^15^ (2008).

Diagnostic criteria of the tested parameters are as follows:

API (Approximal Plaque Index) – hygiene assessment in interdental spaces

100-70% – not proper/69-40% – fair/39-25% – good/<25% optimal

PD (pocket depth) – the distance between the gingiva margin and the pocket bottom

BoP (bleeding on probing) – % of bleeding sites

According to the Offenbacher classification, five clinical conditions of the periodontium are diagnosed:

1. BGI-H – (biofilm-gingival interface: healthy): PD≤3 mm, BoP<10%
2. BGI-G – (biofilm-gingival interface: gingivitis): PD≤3 mm, BoP>10%
3. P1 – (biofilm-gingival interface: deep lesion/low bleeding): PD≥4 mm, BoP<10%
4. P2 – (biofilm-gingival interface: deep lesion/ moderate bleeding): PD≥4 mm, 10%< BoP<50%
5. P3 – (biofilm-gingival interface: deep lesion/severe bleeding): PD≥4 mm, BoP≥50%

Considering the Offenbacher index, two subgroups were created (healthy and gingivitis) and (periodontitis) according to the description of periodontal status.

The study protocol was approved by our research ethics committee (no. KB 623/2015). The patients were informed about the objective of the study, and prior to the clinical assessment, all participants signed an informed consent form to undergo the dental/ periodontal examination. At the end of the clinical part of the study, each patient was informed about his/her oral hygiene status and periodontal status and instructed about the role of proper hygiene and periodontal treatment in the maintenance of oral health.

The Statistica 8.0 software was used to perform statistical analysis. Statistical significance was considered with a p-value<0.05. The parameters of quantitative variables are given as the mean plus standard deviation (SD). Spearman's rank correlation coefficient was used to measure the dependence between the analyzed parameters in the analysis of association.

## Results

Table 1 shows the demographic, clinical, and laboratory parameters of the study patients at the start of the study. The data are shown separately for dentate and edentulous groups.

**Table 1:** Demographic, clinical and laboratory characteristics of hemodialysis patients; quantitative parameters

Parameters	Dentate group (n=103)	Edentulous group (n=25)	p-value
Age (years)	63±13	74±9.0	0.000*
Age at start of hemodialysis	58±14	69±9.0	0.000*
Duration of hemodialysis therapy (months)	52±60	54±51	0.862
Number of teeth	12±7.80	0±0.00	0.000*
Approximal plaque index API (%)	71.55±18.22		
Bleeding on probing index BoP (%)	51.47±13.26		
Pocket depth PD(mm)	3.41±0.80		
Serum C-reactive protein (mg/L)	13.30±14.10	15.80±15.40	0.443
Serum albumin (g/dL)	3.94±0.47	3.91±0.45	0.761
Serum calcium (mg/dL)	8.33±1.06	8.03±2.14	0.306
Serum phosphorus (mg/dL)	5.66±1.64	7.86±11.76	0.067
Serum alkaline phosphatase (UI/L)	107.40±43.20	125.10±83.70	0.138
Parathormone (pg/mL)	567.96±533.91	520.48±425.55	0.68

Results are given as mean±SD;

* p<0.05, statistically significant

The dentate group was composed by 103 patients (43 women/60 men), 63±13 years as the mean age, 52±60 months as the mean time of hemodialysis treatment and 12±7.8 as the mean number of teeth (minimum 1/maximum 27). The edentulous group was composed by 25 patients (18 women/7 men) with an average age of 74±9 years and 54±51 months as the mean time of hemodialysis treatment.

The main causes of chronic kidney disease were: chronic glomerulonephritis (23.5% of patients), diabetic nephropathy (14%), interstitial nephropathy (13%), hypertension (12.5%) and others (37%).

All patients presented secondary hyperparathyroidism. Only the mean age and age of hemodialysis onset were significantly higher in the group of edentulous patients.

There were no significant differences between the groups for the other analyzed parameters.

We found particularly interesting that only one person had a healthy periodontium. The oral hygiene and gingival bleeding parameters were not proper, given that the API index was 71.55±18.22% (minimum 18%/maximum 100%) and the BoP index was also high, with a mean value of 51.47±13.26% (minimum 10%/maximum 87%). The mean PD was 3.41±0.80 mm (minimum 1.75 mm/maximum 6.5 mm); data shown in Table 1.

Estimating the bleeding index and PD according to the Offenbacher index, most of the dentate patients (103 – 62.14%) had gingivitis and 36.9% had moderate or severe periodontitis. The data are shown in Table 2.

**Table 2:** Periodontal status in the dentate group according to the Offenbacher index; qualitative parameters

Offenbacher index	Number of patients	%
Healthy periodontium (H)	1	0.97
Gingivitis (G)	64	62.14
Mild periodontitis (P1)	0	0,00
Moderate periodontitis (P2)	8	7.77
Severe periodontitis (P3)	30	29.13

The comparison of the tested parameters (shown in Table 3) between the subgroups of patients (with gingivitis and periodontitis) showed that the group with a healthier periodontal state (H and G) was significantly younger, started hemodialysis earlier and had twice as many teeth as the group with diagnosed periodontitis (P_2_ and P_3_).

**Table 3:** Demographic, clinical and laboratory characteristics in dentate groups according to the periodontal status; quantitative parameters

Parameters	Healthy periodontium and gingivitis (n=65)	Moderate and severe periodontitis (n=38)	p-value
Age (years)	59±14	69±10	0.000*
Age at start of hemodialysis	55±14	64±11	0.000*
Duration of hemodialysis therapy (months)	50±56	54±67	0.737
Number of teeth	14.77±7.60	7.11±5.57	0.000*
Serum C-reactive protein (mg/L)	12.12±13.49	15.36±15.10	0.264
Serum albumin (g/dL)	3.96±0.51	3.91±0.41	0.601
Serum calcium (mg/dL)	8.30±1.06	8.39±1.06	0.657
Serum phosphorus (mg/dL)	5.87±1.59	5.29±1.68	0.084
Serum alkaline phosphatase (UI/L)	106.56±46.06	108.82±38.42	0.800
Parathormone (pg/mL)	553.99±503.56	591.84±588.48	0.730

Results are given as mean±SD;

* p<0.05, statistically significant

Both subgroups of patients had inflammatory markers (CRP) and calcium-phosphate metabolism (sAP, sP and PTH) parameters above the normal range values and sCa below the normal range – but there were no significant differences between the subgroups.

Spearman's rank correlation coefficient was used for the analyses of association (data in Table 4) and showed that the age at HD onset had a strong positive impact on indices of periodontal status (API index, BoP index and PD) and was negatively correlated with the number of teeth.

**Table 4:** Spearman's rank correlation between the investigated parameters

Parameters	API	BoP	PD	Age (years)	Number of teeth
Age at the start of hemodialysis	0.25*	0.34*	0.41*	0.91*	(-)0.52*
Duration of hemodialysis therapy (months)	-0.05	-0.01	-0.06	0.02	0.00
Serum C-reactive protein (mg/L)	0.07	-0.03	0.09	0.24*	(-)0.23*
Serum albumin (g/dL)	0.05	-0.09	(-)0.20*	(-)0.42*	0.11
Serum calcium (mg/dL)	0.11	-0.09	0.12	0.06	-0.07
Serum phosphorus (mg/dL)	-0.17	-0.15	-0.19	(-)0.26*	0.08
Serum alkaline phosphatase (UI/L)	-0.11	-0.09	0.04	-0.12	0.05
Parathormone (pg/mL)	-0.10	0.00	-0.03	-0.08	0.11

* Correlation coefficients were statistically significant at p=0.05

API: approximal plaque index; BoP: bleeding on probing index; PD: pocket depth

There was also a significant positive correlation between age and level of C-reactive protein, and negative correlations between age and levels of serum albumin and phosphorus.

PD was negatively correlated with serum albumin concentration.

The number of teeth showed strong negative correlation with serum CRP.

## Discussion

This study confirmed the high incidence of poor oral condition in hemodialysis patients^16^.

The main finding of this research is the lack of association between the duration of hemodialysis and severity of periodontitis.

Very few studies, that were performed with a limited number of patients, evaluated the impact of the duration of dialysis on the occurrence of periodontitis in patients, and the findings of some authors are debatable. Cengiz, et al.^16^ (2009), found in a group of 68 HD patients that the periodontal condition worsens as time under dialysis. After 10 years of HD, a much more significant increase was observed. Similarly, Chen, et al.^17^ (2006), assessed 253 HD patients and found that a longer dialysis duration was associated with the severity of periodontitis. On the other hand, the findings from Cengiz, et al.^16^ (2009) and Parkar and Ajithkrishnan^18^ (2012) confirm our observations. In their study, among 304 subjects (152 HD patients/ 152 controls) the results were similar to ours, and also suggested that the higher prevalence of periodontal diseases was mainly caused by oral hygiene negligence, rather than by chronic uremia. Furthermore, Hamissi, et al.^19^ (2009), in a study with 180 Iranian HD patients, noted an unacceptable level of oral hygiene which was not related to the length of time under dialysis.

The most reliable explanation is that the changes occurred much earlier, during the early stages of CKD, in the pre-dialysis period.

From 128 hemodialysis patients, 20% of them were toothless. This number was greater than in the study of Bouattar, et al.^20^ (2011), in which only 11.9% patients were edentulous from a group of 42.

Considering the main objective of our study, we evaluated the demographic characteristics, status of the dentition and the state of periodontal tissues of hemodialysis patients. The mean age of the patients in the dentate group and the edentulous group were 63 years and 74 years, respectively; the mean age at the start of dialysis was 58 and 69 years, respectively. The mean number of teeth in the dentate group was 12, thus, on average, the patients had only 43% of their own teeth. Our results are similar to those from other authors^9^ ^,^ ^20^ ^,^ ^21^. The oral hygiene was not proper, and bleeding indicated a moderate level of inflammation.

According to the Offenbacher index, only one patient had healthy periodontium, most patients had gingivitis and 37% had periodontitis. Patients with gingivitis were younger than those with periodontitis and had twice as many teeth. These results show a better periodontal state when compared to the study of Bhatsange and Patil^4^ (2012). They found gingivitis in 24% and periodontitis in around 75% of the dialysis group patients. Furthermore, the mean PD in our study was 3.41 mm and indicated a moderate stage of periodontitis. In the research of Rodrigues, et al.^21^ (2014), a higher number of patients (59%) had periodontitis and the pocket was deeper on average (4.79 mm). In addition, the study of Schmalz, et al.^22^ (2016), diagnosed moderate periodontitis in 40% and severe periodontitis in 53% of HD patients, periodontal treatment was required in 57% of them.

In our study we found that an older age of patients when starting dialysis was correlated with worse oral hygiene, more severe gingival inflammation and deeper pockets. Additionally, older age was associated with the presence of fewer teeth. Interestingly, Palmer et al.^23^ (2015), in a cohort study with 4205 participants, found that poorer dental health was associated with early death.

For the healthy population, the normal range of CRP level is described as up to 5 mg/L and the range of 5 to 10 mg/L is considered as “high normal”. For both groups. dentate and edentulous, CRP levels were only slightly above a “high normal” level. The data showing almost identical mean CRP levels in the subgroups of patients with gingivitis and periodontitis are particularly noteworthy. In this study, a higher level of serum C-reactive protein was associated with older patients, on the other hand, patients with a lower number of teeth showed a higher C-reactive protein level. These results do not confirm that only oral infection contributes to elevated C-reactive protein levels. The inflammation sources in hemodialysis patients can be multifactorial, and periodontal inflammation seems to be a source of the inflammatory reaction^24^. A similar conclusion is well-known and described in the literature^25^. In the Pallos, et al.^26^ (2015) study, a significantly higher level of salivary CRP was observed in the hemodialysis group, when compared to both the control group and the group with CKD but without HD treatment. The authors concluded that the hemodialysis treatment can influence the CRP level as a result of the release of proinflammatory cytokines. However, the literature also describes that proper periodontal treatment may result in the elimination of periodontal infection and, at the same time, decrease CRP levels^7^.

A lower level of albumin was significantly associated with deeper PD. These results corroborate the suggestions of Rodrigues, et al.^21^ (2014) about a positive association between periodontitis and hypoalbuminemia. Similar findings were also obtained in the research by Chen, et al.^17^ (2006), in which the periodontal status was correlated with malnutrition and inflammation markers. On the other hand, we found no significant difference in serum albumin levels between the dentate group and the edentulous group, or between the group with gingivitis and the group with periodontitis.

However, the negative correlations between age and serum albumin levels in our study group must be noted, it suggests that periodontitis, especially in older patients undergoing hemodialysis, may contribute to malnutrition caused by, among other things, food intake disorders. This finding corroborates previous research^17^.

The serum calcium level did not differ between the dentate and edentulous groups or between groups with gingivitis and periodontitis. Also, no correlation between calcium level and periodontal status was observed. Kong, et al.^27^ (2012), found that hemodialysis patients presented higher serum and salivary phosphorus levels, respectively, in comparison to peritoneal dialysis patients and the healthy group.

There was also no significant correlation between phosphorus level and periodontal state. Finally, only a higher serum level of this microelement in relation to the younger age of patients was observed.

In our HD patients (from both groups), the serum calcium (Ca) level was lower (8.27 mg/dL) in comparison to the results of Kong, et al.^27^ (2012) – (9.3 mg/dL) and Kim, et al.^28^ (2014) – (9.1 mg/dL), and higher than the level (9.29 mg/dL) found in Steven, et al.^29^ (2004). On the other hand, serum phosphorus level (5.63 mg/dL) was lower than in Kong, et al.^27^ (2012), and higher than in the next study cited^28^: 6.3 mg/dL, 5.3 mg/dL and 5.2 mg/dL, respectively.

Another important finding of our study is that, although all patients had secondary hyperparathyroidism (HPT) associated with end-stage renal disease (ESRD – due to the overproduction of PTH), no significant correlation with periodontitis was observed. From our knowledge, there are few observations on this theme in the literature in HD patients. A similar finding was observed by Frankenthal, et al.^30^ (2002) on a small group of 35 HD patients -HPT did not have an appreciable effect on periodontal indices. This further confirms our conclusion that periodontal disease starts in the early stages of ESRD, or already exists at this time.

In our study, the main causes of chronic kidney disease were chronic glomerulonephritis (23.5% of patients), diabetic nephropathy (14%), interstitial nephropathy (13%), hypertension (12.5%) and others (37%). In other studies, the authors described the same common causes of the end-stage renal disease.

However, from the epidemiological point of view, the distribution of CKD causes in our research is not representative. This occurs due to the selection patients for the study – firstly the patient's consent, and in addition the elimination of patients with active inflammation, cancer history, or immunosuppression.

The limitation of our study was the relatively small sample size and the selected cohort of patients.

## Conclusions

In conclusion, our data showed high prevalence and severity of periodontal diseases in hemodialysis patients.

We suggest that there is a high probability that periodontal disease may be present at the early stages of CKD before the hemodialysis treatment started.

Our results reinforce the opinion that oral health promotion, regular dentist assessment, and preventive programs in the period before dialysis can be helpful in changing individual behaviors and reducing the occurrence of irreversible forms of periodontal disease in these particular high cardiovascular risk patients.

Further studies are necessary to assess the potential contribution of periodontal disease to the inflammatory status in hemodialysis patients.
